# Ageing attenuates regional vasoconstriction during acute lowering of upper and lower limbs

**DOI:** 10.1113/EP092882

**Published:** 2025-07-16

**Authors:** John D. Akins, Jeung‐Ki Yoo, Dan‐Dan Sun, Rosemary S. Parker, Marcus A. Urey, Steven A. Romero, Justin S. Lawley, Satyam Sarma, Wanpen Vongpatanasin, Craig G. Crandall, Qi Fu

**Affiliations:** ^1^ Institute for Exercise and Environmental Medicine Texas Health Presbyterian Hospital Dallas Dallas Texas USA; ^2^ Department of Internal Medicine University of Texas Southwestern Medical Center Dallas Texas USA; ^3^ Department of Physiology and Anatomy University of North Texas Health Science Center Fort Worth Texas USA; ^4^ Division of Performance Physiology and Prevention, Department of Sport Science Universität Innsbruck Innsbruck Austria; ^5^ Institute of Mountain Emergency Medicine Eurac Research Bolzano Italy

**Keywords:** ageing, blood pressure regulation, limb dependency, sex differences, venoarteriolar and myogenic response

## Abstract

The venoarteriolar and myogenic response (VMR) is a non‐adrenergic, non‐baroreflex‐mediated mechanism that increases local vascular resistance and contributes to blood pressure (BP) regulation during orthostasis. Despite the importance of the VMR in human cardiovascular control, no information exists elucidating possible differences in the VMR with ageing and sex. We studied 26 healthy young adults [9 male; mean (SD) 28 (4) years old] and 18 healthy older adults [7 male; 71 (3) years old] during acute arm and leg dependency (i.e., limb lowering below heart level) to evoke the VMR. Brachial and femoral artery blood flows were assessed with duplex ultrasound. The VMR was estimated as the percentage reduction in vascular conductance (blood flow/mean arterial BP; in millilitres per minute per millimetre of mercury) from baseline during 5 min of limb dependency. Arm VMR was attenuated in the older versus young adults [−8.7 (1.9)% vs. −26.6 (2.4)%, *p* < 0.001]. Likewise, leg VMR was also attenuated in the older versus young adults [−14.4 (0.8)% vs. −29.1 (2.2)%, *p* < 0.001]. Despite these age‐related differences, there were no sex differences for leg (*p *= 0.096) or arm VMR (*p *= 0.825). These data suggest that ageing attenuates the VMR in both the upper and lower limbs, but sex does not impact these responses. This phenomenon might contribute to altered BP regulation in older adults, either protecting against excessive orthostatic BP elevations or contributing to orthostatic hypotension. Future research is needed to determine the mechanisms of an attenuated VMR with ageing and whether these findings extend to populations with hypertension and cardiovascular/metabolic disease.

## Abstract (highlights)

- **What is the central question of this study?**
  The venoarteriolar and myogenic response (VMR) contributes to blood flow and pressure regulation, particularly during orthostasis. Despite its importance in cardiovascular control, the impacts of ageing and sex on the VMR remain unclear.
- **What is the main finding and its importance?**
  Healthy older adults, relative to young adults, exhibit an attenuated VMR in both the arm and the leg during acute limb lowering, with no sex‐specific differences in either age group. This reduced VMR might either protect against excessive rises in blood pressure during orthostasis or contribute to orthostatic hypotension in older adults.

## 1 INTRODUCTION

An increase in blood pressure (BP) with ageing remains common in adults (Martin et al., 2025). Although the aetiology of impaired BP regulation with ageing is multifactorial, increasing vascular tone from various sources can contribute (Esler, 2000; Laitinen et al., 2004; Lakatta, 1993; Lund‐Johansen, 1991; Shannon et al., 1991), particularly as a byproduct of arterial stiffening (Heagerty et al., 2010). As such, understanding the physiological mechanisms regulating altered vascular tone with ageing helps to identify pathways that impact BP regulation.

Beyond the typically cited increase in efferent sympathetic outflow (Esler, 2000; Keir et al., 2020; Matsukawa et al., 1998), local mechanisms can also drive increases in vascular resistance. Derived from a combination of a local venoarteriolar axon reflex (i.e., venoarteriolar response) secondary to venous congestion (i.e., pressure of >25 mmHg) and a myogenic response (Gaskell & Burton, 1953; Henriksen, 1976b; Henriksen & Sejrsen, 1976, 1977; Okazaki et al., 2005), the combined venoarteriolar and myogenic responses (VMRs) elicit potent local vasoconstriction independent of adrenergic mechanisms (Crandall et al., 2002). The VMR effectively induces vasoconstriction in individuals with spinal cord injury (Groothuis et al., 2005; Trbovich et al., 2022) or epidural blockade (Henriksen et al., 1983; Skagen et al., 1982) during orthostasis. However, to date, there are no data specifically investigating age‐induced changes in the VMR, although data suggest that the VMR is impaired in disease states such as diabetes and peripheral artery disease (Belcaro et al., 1989; Cisek et al., 1997; Wahlberg et al., 1990). On this point, given that ageing is associated with an increased prevalence of hypertension (Martin et al., 2025) and that conditions such as hypertension either alter or are influenced by (mal)adaptive BP‐regulating mechanisms (Grassi et al., 2015; Mancia & Grassi, 2014; Schiffrin, 2012), it is imperative to study the VMR during healthy ageing. Furthermore, given the apparent sex differences in autonomic cardiovascular control (Matsukawa et al., 1998; Narkiewicz et al., 2005; Stanhewicz et al., 2018; Taylor et al., 2020) and cardiovascular disease risk, particularly with ageing (Martin et al., 2025), it is important to understand how the VMR might also differ between men and women.

In this study, therefore, we aimed to assess the VMR during leg and arm dependency (i.e., acute lowering of the limb below the level of the heart) in otherwise healthy young and older men and women. We hypothesized that older adults would demonstrate a greater VMR than younger adults and that women would have a lower VMR than men.

## 2 MATERIALS AND METHODS

### 2.1 Ethical approval

All procedures for this study were approved by the Institutional Review Boards at the University of Texas Southwestern Medical Center and Texas Health Presbyterian Hospital Dallas (STU 112015‐099). Participants were provided with a verbal and written description of all purposes, procedures and risks associated with the study before providing their informed, written consent. This study conformed to the *Declaration of Helsinki*, except for registration in a database.

### 2.2 Participants

Twenty‐six (17 women and 9 men) young and 18 (11 women and 7 men) older adults participated in this study. Participants were free of overt cardiovascular and other major clinical diseases, including hypertension, as assessed by medical history, brief physical examination, 12‐lead ECG and 24 h ambulatory BP assessment. Additionally, all were non‐smokers and at most recreationally active. Young women were evaluated in the mid‐luteal phase of the menstrual cycle or during the high‐hormone phase of contraceptive use (oral combined, *n* = 5; vaginal ring, *n* = 1; hormonal intrauterine device, *n* = 1; copper intrauterine device, *n* = 1). Older women were all postmenopausal, and none of the older adults had taken hormone replacement therapy within the last 5 years.

### 2.3 Study protocol

Experiments were performed in the morning ≥2 h after a light breakfast and ≥24 h after the last caffeine or alcohol intake. Each participant lay supine on a patient bed in a quiet, temperature‐controlled (∼23°C–25°C) laboratory. Participants were instrumented for the measurement of heart rate and BP. Following ≥20 min of supine rest, the VMR assessment was performed, whereby the order of limb dependency was randomized. Following baseline assessment of BP, heart rate, and artery diameter and blood velocity, the arm or leg was lowered below heart level at an angle of ∼30°. This position corresponded to a displacement below heart level of 43 (5) cm for the leg and 40 (4) cm for the arm as measured at the end of the foot and hand, respectively. After 60 s of stabilization, the same cardiovascular and haemodynamic measurements were completed for 4 min with the limb in the dependent position before the arm or leg was returned to the horizontal position. This procedure was then repeated for the remaining limb.

### 2.4 Doppler ultrasound measures

Common femoral and brachial artery diameters and blood velocities were collected using duplex ultrasonography (LOGIQ e, GE Healthcare, Chicago, IL, USA) equipped with an adjustable‐frequency linear array transducer. Briefly, the common femoral artery was imaged ∼2 cm proximal to the femoral artery bifurcation and optimized for clear delineation of the lumen and vessel walls through B‐mode. Likewise, the brachial artery was imaged approximately midway between the shoulder and antecubital fossa. Duplex mode was then used for simultaneous capture of artery diameter and blood velocity. The sample volume was set to encompass the entire lumen, without extending into the surrounding tissue, at an insonation angle of ≤60°. Screen images were recorded using commercially available software (Camtasia, TechSmith Corporation, East Lansing, MI, USA), and the Doppler ultrasound audio output was interfaced with a computer running custom software to capture blood velocity (Buck et al., 2014; Romero et al., 2017). Artery diameter was measured using a commercially available edge‐detection and wall‐tracking software package (Brachial Analyzer, Medical Imaging Applications, LLC, Coralville, IA, USA). Data analysis was performed by the same investigator (J.‐K.Y.), who was blinded to subject identity and group.

### 2.5 Cardiovascular measurements

Heart rate was measured from lead II of the ECG (Dash 4000, GE HealthCare, Chicago, IL, USA), and intermittent brachial BP was measured by electrosphygmomanometry (model 4240, SunTech Medical Instruments, Morrisville, NC, USA) from the non‐lowered arm. Beat‐by‐beat systolic and diastolic BP were derived from finger photoplethysmography in the non‐lowered arm (BMEYE, Nexfin, Amsterdam, The Netherlands).

### 2.6 Data analysis

Cardiovascular data were sampled at 500 Hz using a commercial data acquisition system (AcqKnowledge, Biopac Systems, Santa Barbara, CA, USA). Data from the last 60 s of the baseline were averaged and compared with the limb dependency conditions. For limb dependency, data were obtained starting 1 min after limb lowering and collected continuously until termination of the test. Data for this period are presented as 60, 120, 180 and 240 s, corresponding to the 60–120, 120–180, 180–240 and 240–300 s epochs following limb dependency. The BP, artery diameter and artery mean blood velocity were averaged in 60 s bins and used for the subsequent calculation of blood flow [mean blood velocity × π × (0.5 × artery diameter)^2^ × 60; in millilitres per minute] and vascular conductance (blood flow/mean arterial BP; in millilitres per minute per millimetre of mercury). The percentage reduction in vascular conductance during limb dependency was used to assess the VMR (Brothers et al., 2009; Okazaki et al., 2005).

### 2.7 Statistical analysis

Participant characteristics and baseline vascular data were compared using Student's unpaired, two‐tailed *t*‐tests with Welch's correction or Mann–Whitney *U*‐tests, where appropriate. Cardiovascular and haemodynamic data during limb dependency were analysed through linear mixed‐effects models, with the fixed factors of age group and VMR time, using a diagonal covariance matrix and fitted using restricted maximum likelihood, whereby the absolute blood flow and conductance data were also analysed using baseline as a covariate. The peak change data for leg and arm VMRs were compared using two‐way ANOVA, with the fixed factors of age group and sex. In the event of significant main effects or interactions, *post hoc* Šidák multiple comparisons were made. All data were inspected for normality. Effect sizes for *t*‐tests were assessed using Cohen's *d* and for Mann–Whitney *U*‐tests using rank biserial correlation (i.e., *r*). Effect sizes for linear mixed models were estimated as η^2^_p_ using the *effectsize* package developed for R and made available as a web application (Ben‐Shachar et al., 2020). Small (*d* = 0.2, *r* = 0.1, η^2^_p_ = 0.01), medium (*d* = 0.5, *r* = 0.3, η^2^_p_ = 0.06) and large (*d* = 0.8, *r* = 0.5, η^2^_p_ = 0.14) effects were established for the primary analyses. All analyses were completed using SPSS v.20 (IBM Corp., Armonk, NY, USA), Prism 10 (GraphPad Software Inc., San Diego, CA, USA) or jamovi v.2.6 (The jamovi project; Sydney, NSW, Australia). Statistical significance was set a priori at α = 0.05. Values are expressed as means (SD) unless otherwise noted.

## 3 RESULTS

### 3.1 Participant characteristics

Participant characteristics are reported in Table 1. Beyond the designed difference in age (*p* < 0.001), the groups were matched for anthropometrics and awake BP (*p* range: 0.090–0.754).

**TABLE 1:** Participant characteristics.

Characteristic	Young	Older	*p*‐Value	Effect size
Male/female	9/17	7/11	–	–
Age, years	28 (4)	71 (3)	<0.001	10.874
Height, m	1.70 (0.10)	1.64 (0.09)	0.090	0.528
Body mass, kg	67.9 (13.4)	64.5 (11.6)	0.372	0.273
Body mass index, kg m^−2^	23.5 (3.1)	23.8 (3.2)	0.754	0.096
Awake ambulatory SBP, mmHg	122 [7]	123 [5]	0.322	0.111
Awake ambulatory DBP, mmHg	73 [8]	71 [4]	0.255	0.218
Self‐reported race/ethnicity (*n*)				
Asian	7	6	–	–
Hispanic	3	0	–	–
Non‐Hispanic Black	3	2	–	–
Non‐Hispanic White	13	10	–	–

*Note*: Data are presented as *n*, means (SD) for normally distributed data, or median [interquartile range] for non‐normally distributed data. Normally distributed data were compared using Student's unpaired *t*‐tests with Welch's correction and Cohen's *d*, whereas non‐normally distributed data were compared using Mann–Whitney *U*‐tests and rank biserial correlation.

Abbreviations: DBP, diastolic blood pressure; SBP, systolic blood pressure.

### 3.2 Baseline arterial haemodynamics

Baseline measurements of femoral and brachial artery vessel diameter, mean blood velocity, mean blood flow and vascular conductance are reported in Table 2. None of the femoral artery measurements were different between the young and older participants (*p* range: 0.193–0.877). Brachial artery diameter, mean blood velocity and mean blood flow were not different between the young and older participants (*p* range: 0.105–0.961). However, mean brachial vascular conductance was lower in the older adults (*p* = 0.036).

**TABLE 2:** Baseline arterial haemodynamics.

Parameter	Young	Older	*p*‐Value	Effect size
Femoral artery				
Vessel diameter, mm	7.9 [1.6]	7.9 [1.4]	0.765	0.056
Mean blood velocity, cm s^−1^	6.20 [4.33]	7.50 [1.98]	0.193	0.235
Mean blood flow, mL min^−1^	219.0 (90.8)	236.3 (95.8)	0.552	0.185
Vascular conductance, mL min^−1^ mmHg^−1^	2.78 [1.58]	2.30 [1.20]	0.877	0.030
Brachial artery				
Vessel diameter, mm	3.6 (0.7)	3.6 (0.7)	0.961	0.015
Mean blood velocity, cm s^−1^	5.81 [2.93]	5.19 [2.00]	0.252	0.207
Mean blood flow, mL min^−1^	38.9 (19.0)	31.2 (11.5)	0.105	0.487
Vascular conductance, mL min^−1^ mmHg^−1^	0.48 (0.22)	0.36 (0.13)	0.036	0.640

*Note*: Data are presented as means (SD) for normally distributed data or median [interquartile range] for non‐normally distributed data. Normally distributed data were compared using Student's unpaired *t*‐tests with Welch's correction and Cohen's *d*, whereas non‐normally distributed data were compared using Mann–Whitney *U*‐tests and rank biserial correlation.

### 3.3 Baseline and limb dependency cardiovascular outcomes

Heart rate in the young participants increased from baseline during leg dependency [61 (12) vs. 63 (12) beats min^−1^; *p* = 0.005], but not during arm dependency [62 (12) vs. 62 (12) beats min^−1^; *p* = 0.301]. In the older participants, heart rate did not change from baseline during either leg dependency [62 (9) vs. 63 (8) beats min^−1^; *p* = 0.409] or arm dependency [61 (8) vs. 61 (7) beats min^−1^; *p* = 0.740].

Mean arterial BP in the young participants did not change from baseline to leg dependency [78 (7) vs. 79 (5) mmHg; *p* = 0.332] or arm dependency [80 (6) vs. 79 (6) mmHg; *p* = 0.116]. Contrarily, the older participants experienced a reduction in mean arterial BP from baseline during leg dependency [87 (8) vs. 85 (8) mmHg; *p* = 0.007], which did not manifest during arm dependency [86 (7) vs. 84 (6) mmHg; *p* = 0.080].

### 3.4 Influence of age on VMRs

**FIGURE 1:**
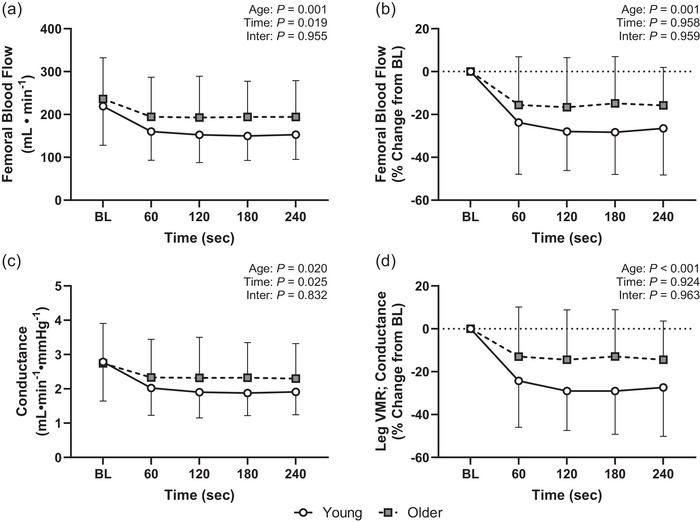
Leg VMRs during limb dependency in young (*n* = 26) and older (*n* = 18) participants. Lowering of the leg elicited reductions in femoral blood flow [absolute (a) and relative change from BL (b)] and vascular conductance [absolute (c) and relative change from BL (d)], which were also different between the young and older participants. Data are presented as means (SD) and analysed using linear mixed‐effects models. Abbreviations: BL, baseline; VMR, venoarteriolar and myogenic response.

Leg dependency produced the expected reductions in both femoral artery blood flow and vascular conductance in both the young and older participants (Figure 1). The absolute reduction in femoral blood flow (*p* = 0.001, η^2^_p_ = 0.052; Figure 1a) and femoral vascular conductance (*p* = 0.020, η^2^_p_ = 0.027; Figure 1c) was attenuated in the older participants. These findings for femoral blood flow and vascular conductance remained after covarying for the respective baseline value (both *p* < 0.001). Likewise, when expressed as a percentage change from baseline, the reduction in femoral blood flow (*p* = 0.001, η^2^_p_ = 0.064; Figure 1b) and femoral vascular conductance (i.e., leg VMR; *p* < 0.001, η^2^_p_ = 0.096; Figure 1d) remained attenuated in the older participants.

**FIGURE 2:**
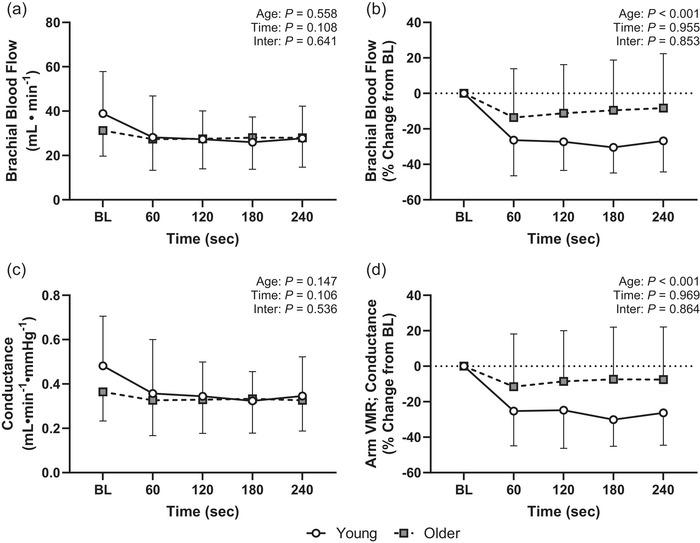
Arm VMRs during limb dependency in young (*n* = 26) and older (*n* = 18) participants. Although lowering of the arm did not elicit significant reductions in brachial blood flow [absolute (a) and relative change from BL (b)] or vascular conductance [absolute (c) and relative change from BL (d)], the relative change from baseline for both variables was greater in the young versus older participants. Data are presented as means (SD) and analysed using linear mixed‐effects models. Abbreviations: BL, baseline; VMR, venoarteriolar and myogenic response.

Interestingly, arm dependency did not reduce brachial artery blood flow or brachial vascular conductance (Figure 2), and there were no differences between the young and older participants for the absolute reductions in either variable between groups (flow: *p* = 0.558, η^2^_p_ = 0.002; Figure 2a; conductance: *p* = 0.147, η^2^_p_ = 0.011; Figure 2c). This lack of difference remained after covarying for baseline for both brachial blood flow (*p* = 0.927) and vascular conductance (*p* = 0.693). However, when expressed as a percentage reduction from baseline, the older participants had an attenuated reduction in brachial blood flow (*p* < 0.001, η^2^_p_ = 0.129; Figure 2b) and brachial vascular conductance (i.e., arm VMR; *p* < 0.001, η^2^_p_ = 0.123; Figure 2d).

### 3.5 Influence of sex on VMRs

**FIGURE 3:**
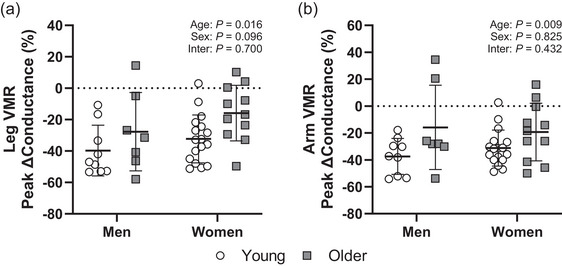
Peak leg and arm VMRs during leg dependency (a) and arm dependency (b) in young men (*n* = 9) and women (*n* = 17) and older men (*n* = 7) and women (*n* = 11). No differences were found in the peak leg or arm VMR between men and women in either group, despite a reduced VMR with ageing. Data are presented as means (SD) with individual values and analysed using two‐way ANOVA. Abbreviation: VMR, venoarteriolar and myogenic response.

There were no differences between men and women for leg or arm VMR (Figure 3). Specifically, although the peak change in leg VMR was greater in the older participants (*p* = 0.016, η^2^_p_ = 0.136; Figure 3a), this response was not different between the men and women (*p* = 0.096, η^2^_p_ = 0.068). Likewise, the peak change in arm VMR was greater in the older participants (*p* = 0.009, η^2^_p_ = 0.158; Figure 3b), but was not different between the men and women (*p* = 0.825, η^2^_p_ = 0.001). There was no age‐by‐sex interaction for leg VMR (*p* = 0.700, η^2^_p_ = 0.004) or arm VMR (*p* = 0.432, η^2^_p_ = 0.016).

## 4 DISCUSSION

In this study, we found that older adults had a reduced leg and arm VMR relative to young adults, yet without sex differences within each group. As such, these data suggest that healthy ageing, but not sex, attenuates the VMR in both the lower and upper limbs, although the latter observation might be influenced by baseline blood flow or vascular conductance. Accordingly, an attenuated VMR might possess clinical relevance in the maintenance of ambulatory BP with healthy ageing by preventing excessive vasoconstriction and exaggerated BP increases during orthostasis. However, the attenuated VMR might also contribute to an increased prevalence of orthostatic hypotension in older adults (Ricci et al., 2015).

### 4.1 Mechanisms regulating the VMRs

The rise in arteriolar and venous transmural pressures that occurs during limb dependency activates various signalling pathways that elicit arteriolar vasoconstriction, therefore limiting the rise in capillary pressure (Crandall et al., 2002; Henriksen, 1976b; Henriksen & Sejrsen, 1976, 1977; Henriksen et al., 1983; Okazaki et al., 2005; Skagen & Bonde‐Petersen, 1982; Vissing et al., 1997). Importantly, the VMR is independent of sympathetic adrenergic transmission (Crandall et al., 2002), yet sensitive to the application of local anaesthetics (Crandall et al., 2002; Henriksen & Sejrsen, 1977; Okazaki et al., 2005; Skagen & Henriksen, 1983). The VMR also acts independently of the baroreflex and, thus, should not cause substantial changes in heart rate and BP. We did observe, however, a significant increase in heart rate during leg dependency in the young participants and a significant reduction in mean arterial BP during leg dependency in the older participants. That said, these changes were small (∼2 beats min^−1^ and ∼2 mmHg, respectively), and the opposite variable (e.g., BP and heart rate, respectively) for these groups remained unchanged. Thus, we believe that the baroreflex had minimal influence on our findings. As such, its independence as a non‐adrenergic, non‐baroreflex‐related mechanism allows the VMR to regulate vascular tone and blood flow locally.

### 4.2 Attenuated venoarteriolar myogenic responses with ageing

Typically, with ageing, the progressive increase in BP is mirrored by an increase in muscle sympathetic nerve activity, whereby there also appears to be a sex difference in the rate of rise in sympathetic outflow with ageing, which is greater in women than in men (Matsukawa et al., 1998; Narkiewicz et al., 2005). As such, the rise in BP with ageing appears to be a byproduct of a greater reliance on increased vascular resistance secondary to sympathetic outflow, particularly during orthostasis (Laitinen et al., 2004; Shannon et al., 1991). However, some of the increase in systemic vascular resistance during orthostasis might be a product of local regulation of vascular resistance (i.e., the VMR). Indeed, the VMR is postulated to account for ∼45% of the vasoconstrictor response during orthostasis (Henriksen, 1976a; Henriksen & Sejrsen, 1976, 1977; Skagen, 1982; Skagen et al., 1985) and remains intact in patients with spinal cord injury (Groothuis et al., 2005; Trbovich et al., 2022). As such, the respective contributions of the VMR might play a direct role in regulating ambulatory BP, particularly with ageing.

Interestingly, our results demonstrate no differences in awake ambulatory BP between our young and older participants despite the attenuated arm and leg VMR in the older adults. During an orthostatic challenge, similar adaptations occur, whereby a greater reliance on sympathetically mediated vascular resistance offsets a reduced reliance on cardiac output, which normalizes BP responses (Laitinen et al., 2004; Shannon et al., 1991). Thus, although speculative, this reduced VMR might serve as a protective mechanism preventing exaggerated increases in systemic vascular resistance during orthostasis despite greater sympathetic outflow in older adults. Alternatively, a reduced VMR might contribute to an observed greater prevalence of orthostatic hypotension in older adults (Ricci et al., 2015). Given the reduced cardiac output response and greater reliance on sympathetic vasoconstriction in older adults to orthostasis, a reduced VMR might leave these individuals prone to greater reductions in BP during orthostasis, especially during initial postural transitions. That said, several studies have reported varying BP responses in older adults, including increases (Badrov et al., 2020; Barantke et al., 2008) and decreases in BP during orthostasis (Barnett et al., 1999; Mellingsæter et al., 2013). Thus, further studies are warranted to determine the respective contributions of sympathetic outflow and the VMR to peripheral vascular resistance during orthostasis and limb dependency, particularly with respect to ageing.

Additionally, our findings might implicate attenuations in myogenic tone or sensory nerve activity with ageing. Previous work in rats demonstrated reduced myogenic responses in soleus and gastrocnemius arterioles (Muller‐Delp et al., 2002), secondary to changing contributions of large‐conductance Ca^2+^‐activated and voltage‐dependent K^+^ channels (Kang et al., 2009). Additional evidence suggests a reduction in sensory nerve function with ageing in both human and rodent models (Helme & McKernan, 1985; Khalil et al., 1997). Thus, these combined effects might contribute to the reduced VMR with ageing in the present study.

### 4.3 Lack of sex differences in the venoarteriolar myogenic response

Sex differences in vascular function across the lifespan have been reviewed extensively and are typically attributed to differences in sex hormones and their interactions with their vascular receptors (Green et al., 2016; Pabbidi et al., 2018; Sader & Celermajer, 2002; Stanhewicz et al., 2018). Thus, it stands to reason that the VMR might also be affected by sex, resulting in differing VMR responses between men and women, particularly with ageing. However, we did not observe any sex‐specific differences in the VMR in either limb in our young or older participants. This response might have important implications for the mechanisms governing orthostatic BP between sexes, especially given that young women typically have reduced orthostatic tolerance (Fu et al., 2004), whereas older women have greater orthostatic tolerance than age‐matched men (Mellingsæter et al., 2013).

Importantly, the young women were tested during a high‐hormone phase, and thus there was a greater likelihood to determine whether sex hormones have a role in modulating the VMR during limb dependency. That said, given that the same participants were not tested during the low‐hormone phase, it remains unclear whether the VMR changes across the menstrual cycle or different phases of hormonal contraceptive use. A previous meta‐analysis, however, identified that the menstrual cycle might have little to no impact on microvascular and vascular smooth muscle function (Williams et al., 2020). A further review also noted that, although the literature remains sparse, there appears to be no impact of hormonal contraception on macro‐ or microvascular smooth muscle function, although endothelial function does appear to be influenced (Williams & MacDonald, 2021). Additionally, given that there were no sex differences in our older cohort, it is less likely that sex hormones influence the VMR, although any offsetting effects of natural ageing and sex hormone loss on the VMR remain unknown.

### 4.4 Experimental considerations

Although our study demonstrates unique vasomotor responses to limb dependency with ageing, some experimental considerations should be noted. First, although the VMR is subject to several complex mechanisms, we did not address these pathways. Thus, although we have provided some speculatory evidence of mechanisms that might be regulating the attenuated venoarteriolar response with ageing (e.g., large‐conductance Ca^2+^‐activated and voltage‐dependent K^+^ channels), further studies are needed to elucidate fully the impact of ageing on the VMR.

Second, we did not separately evaluate the venoarteriolar and myogenic components of the VMR. Given that previous work has proposed varying contributions of each component in the leg (venoarteriolar, 45%; myogenic, 55%) and arm (venoarteriolar, 58%; myogenic, 42%) during limb dependency (Okazaki et al., 2005), the use of an additional model (e.g., venous cuff occlusion) might allow for better interrogation of the separate venoarteriolar and myogenic components.

Third, although a majority of the baseline arterial haemodynamic data were not different between the two groups, we cannot rule out the possibility that baseline blood flow and conductance did not influence our findings, particularly in the brachial artery. As we demonstrated, arm VMR was not different between groups after covarying for baseline despite a difference when expressed as a percentage reduction from baseline. As such, recreating these findings in conditions when baseline blood flow and conductance are matched would help to corroborate our observations of an attenuated VMR in older adults and confirm whether there are limb‐specific differences.

Fourth, we included only relatively healthy individuals in this study, hence our findings might not be applicable to populations with various disease states. Conditions such as diabetes and peripheral artery disease can impair the VMR (Belcaro et al., 1989; Cisek et al., 1997; Wahlberg et al., 1990). Thus, our findings provide the impetus to study the VMR in various patient populations, including those with hypertension and overt cardiovascular/metabolic diseases.

Fifth, we did not measure sympathetic outflow or orthostatic stress responses (e.g., head‐up tilt) in these participants. Although we speculate that the reduced VMR might help to offset greater sympathetic outflow in older individuals, particularly during orthostasis (Ng et al., 1995), we cannot be sure of this with the present protocol. Although measuring sympathetic outflow and vascular resistance during limb dependency and orthostasis would provide greater insight into the contribution by the VMR to vasoconstriction, the use of orthostasis also recruits other reflexes (e.g., baroreflex, vestibulo‐sympathetic reflex). As such, a more invasive approach, such as a neural blockade or drug infusion, would be required to isolate the VMR in these conditions (Henriksen et al., 1983; Kooijman et al., 2009; Skagen et al., 1982).

Finally, and related to the lack of measurement of sympathetic outflow, we also did not take measurements of venous congestion in this study. Given that previous work demonstrates that venous distension evokes reflex increases in sympathetic outflow (Cui et al., 2012), it is possible that limb dependency might have induced these conditions. However, venous congestion during limb dependency does not appear to induce an increase in sympathetic outflow, as evidenced by the minimal BP and heart rate changes in the present study. Additionally, during orthostatic stress, the increases in arterial and venous pressures are fairly similar, albeit with a smaller change in capillary pressure, which maintains the transcapillary pressure (de Graaff et al., 2003). Thus, the reductions in arterial blood flow and conductance might not be a byproduct of altered transcapillary pressure secondary to venous pooling, but rather the vasomotor responses of the VMR.

## 5 CONCLUSION

We observed, for the first time, that healthy ageing contributes to an attenuated VMR in both the leg and arm during limb dependency, although with a potential influence of baseline blood flow in the arm. However, despite previous reports of sex differences in vascular function, we did not observe differences in the VMR between young or older men and women. A reduced VMR might offset other factors that increase vascular resistance (e.g., sympathetic nerve activity), thus protecting BP regulation during orthostasis in older adults. Alternatively, this reduced VMR might contribute to a greater rate of orthostatic hypotension in older adults. Future studies are needed to assess whether the VMR is changed further by disease states in older adults, including hypertension, to gain a better understanding of their possible synergistic roles with ageing.

## AUTHOR CONTRIBUTIONS

Qi Fu, Craig G. Crandall and Wanpen Vongpatanasin conceived and designed the study. Jeung‐Ki Yoo, Dan‐Dan Sun, Marcus A. Urey, Rosemary S. Parker and Qi Fu performed data acquisition. John D. Akins, Jeung‐Ki Yoo, Steven A. Romero, Justin S. Lawley, Satyam Sarma and Qi Fu helped to analyse and interpret the data. Satyam Sarma provided on‐site medical supervision for the experiments. John D. Akins prepared the figures and drafted the manuscript. John D. Akins, Jeung‐Ki Yoo, Dan‐Dan Sun, Rosemary S. Parker, Marcus A. Urey, Steven A. Romero, Justin S. Lawley, Satyam Sarma, Wanpen Vongpatanasin, Craig G. Crandall and Qi Fu edited and revised the manuscript. All authors approved the final version of the manuscript and agree to be accountable for all aspects of the work in ensuring that questions related to the accuracy or integrity of any part of the work are appropriately investigated and resolved. All persons designated as authors qualify for authorship, and all those who qualify for authorship are listed.

## CONFLICT OF INTEREST

None declared.

## Data Availability

Data will be made available by the corresponding author upon reasonable request.
